# Investigating Adolescents’ Video Gaming and Gambling Activities, and Their Relationship With Behavioral, Emotional, and Social Difficulties: Protocol for a Multi-Informant Study

**DOI:** 10.2196/33376

**Published:** 2022-02-25

**Authors:** Loredana Cena, Matteo Rota, Alice Trainini, Sara Zecca, Sofia Bonetti Zappa, Nella Tralli, Alberto Stefana

**Affiliations:** 1 Department of Clinical and Experimental Science University of Brescia Brescia Italy; 2 Department of Molecular and Translational Medicine University of Brescia Brescia Italy; 3 Department of Brain and Behavioural Sciences University of Pavia Pavia Italy

**Keywords:** adolescents, gaming disorder, gambling disorder, pathological video gaming, pathological gambling

## Abstract

**Background:**

Growing empirical evidence suggests that adolescents have a relatively greater propensity to develop problematic video gaming or gambling habits.

**Objective:**

The main objectives of this study are to estimate the prevalence of potential pathological gambling and video game use among adolescent students and to evaluate their risk factors.

**Methods:**

This is a cross-sectional multi-informant study based on an online survey. It will include a sample of adolescents attending secondary schools located in Brescia, northern Italy, their schoolteachers, and parents. The survey includes extensive data on adolescents’ (1) demographic, social, economic, and environmental characteristics; (2) behavioral, emotional, and social problems and adaptive functioning; (3) emotional and social loneliness; (4) perception of the reasons to use social networks; (5) video game habits and pathological use of video gaming; and (6) gambling behaviors.

**Results:**

This protocol was approved by the Institutional Ethics Board of the Spedali Civili of Brescia (Italy). We expect to collect data from 793 or more adolescent students, as determined by our sample size calculation.

**Conclusions:**

This multisite project will make a substantial contribution to (1) the implementation of a system for identifying pathological gambling and pathological video game use among adolescents, allowing for interventions aimed at improving adolescents’ financial, emotional, and social well-being; and (2) the identification of distinct profiles of gamblers and pathological video gamers that will contribute to setting up effective targeted prevention measures. Understanding the causes and impact of gambling and pathological video gaming on adolescents is a public health issue.

**International Registered Report Identifier (IRRID):**

DERR1-10.2196/33376

## Introduction

### Background

Adolescence is characterized by increased risk-taking behaviors [1]. Consequent to these behavioral alterations is the adolescents’ relatively greater propensity to develop problematic video gaming or gambling habits, which in recent years has become a serious social and public health policy issue [2-4]. In Europe, where a legalization and liberalization of gambling markets has taken place over the past few decades [5], adolescent gambling prevalence rates range from 0.2% to 12.3% [2], whereas the prevalence of internet gaming disorder rates range from 1.2% to 5.0% [6]. Such large variances reflect the difficulties in obtaining accurate measurements of this phenomenon.

Video gaming is very popular among adolescents [7] and can be associated with many positive aspects of youth development [8,9]. However, during recent years, an increasing number of adolescents are overusing video games that could lead to a gaming disorder, defined as “a pattern of persistent or recurrent gaming behavior (‘digital gaming’ or ‘video gaming’) […] manifested by: 1) impaired control over gaming […]; 2) increasing priority given to gaming to the extent that gaming takes precedence over other life interests and daily activities; and 3) continuation or escalation of gaming despite the occurrence of negative consequences” [10]. Indeed, a recent review found that the prevalence of internet gaming disorder [11] ranges from 1.2% to 5.0% in the European Union [6].

Growing empirical evidence suggests that gaming disorder is associated with internalized (such as anxiety and depression) and externalized (such as attention deficit hyperactivity disorder) symptoms [12-14], substance use [15,16], and physical violence including weapon carrying and use [17]. Furthermore, video gaming problems seem to be a gateway to problematic gambling behavior [18]. This is not, however, surprising when you consider that 7 out of 9 Diagnostic and Statistical Manual of Mental Disorders-5 (DSM-5) [14] criteria for internet gaming disorder are identical to those used for diagnosing gambling disorder [19].

Although the majority of adolescents who have had experience of gambling maintain recreational and controlled activities, some of them develop a gambling disorder. This latter is characterized by “a pattern of persistent or recurrent gambling behavior, which may be online [...] or offline, manifested by: 1) impaired control over gambling […]; 2) increasing priority given to gambling to the extent that gambling takes precedence over other life interests and daily activities; and 3) continuation or escalation of gambling despite the occurrence of negative consequences” [10]. Like gaming disorders, gambling disorder is associated with various mental health issues, such as high levels of impulsivity, anxiety, depression, and stress [20-22] as well as substance use [23].

Few empirical research studies have explored both problematic gaming and gambling habits, the relationship between them, and their effects on adolescents’ well-being, often considering a limited number of characteristics of adolescents’ psychological, interpersonal, and environmental lives [24,25].

Italian studies have shown that being male, poor parental monitoring, having an anomic view of the social environment [26], using more than 1 game (especially strategy games), having a gambler father or both parents who used to gamble [27], living with nonbirth parents, and having a higher perception of the family’s financial status [28] are factors that increase the possibility of becoming at risk of problem gamblers. These studies investigate the Italian profile of adolescent and young adult gamblers.

### Objectives

The main aims of this study are to:

- estimate the prevalence of potential pathological gambling and pathological video game use among a sample of adolescent students attending upper-secondary schools;
- estimate the prevalence of emotional/behavioral problems in a sample of adolescent students attending upper-secondary schools, evaluating the correlation between data obtained from different informants (adolescents, parents, and teachers);
- identify distinct profiles of gamblers and pathological video gamers based on their self-reported gambling/gaming behaviors;
- explore risk factors of pathological gambling and pathological video game use among adolescent students attending upper-secondary schools;
- develop a model for identifying adolescents’ pathological gambling and pathological video game use.

## Methods

### Study Design and Setting

This is a cross-sectional multi-informant study conducted in the province of Brescia, the second most populous city in Lombardy (northern Italy), among adolescent students attending secondary schools, their schoolteachers, and parents. The study will involve a total of 5 first-grade secondary schools (“middle school”; aged 11-14 years) and 5 second-grade secondary schools (“high school”; aged 14-19 years).

### Pretest

A pretest will be performed to validate the feasibility of the study before beginning data collection. Specifically, we recruited a convenience sample of 28 students to test the comprehensibility of the survey questionnaires and the correct functioning of the online survey system.

### Recruitment

We will recruit participants from 10 schools (5 middle schools and 5 high schools). We will present information about the research study both orally and in written form to the schoolteachers of each school. Subsequently, the research team members will visit each participating school to provide a seminar with the aim of presenting the study to students, and then contact the parents or guardians to obtain informed consent for their children to participate in the study. The final enrollment is expected to be 793 adolescents.

### Eligibility Criteria

Inclusion criteria are that the participants are attending the third year of middle school or any of the 5 years of high school. Furthermore, the participation in the study of both a parent (or guardian) and a schoolteacher is required for each student. Students are also required to have adequate knowledge in Italian language to be able to complete the questionnaire.

### Typically Developing Reference Sample

Adolescents are eligible to participate in the reference sample if they are students aged between 13 and 19 years, have acquired Italian language skills, and do not have an intellectual disability.

### Data Collection

The participants will complete a 1-time online survey between February and October 2020. We will collect data from multiple informants, including students’ self-report questionnaires and parents’ (or guardians’) and schoolteachers’ questionnaires on their child/pupil (Table 1). Each participant will complete questionnaires using an online survey tool (LimeSurvey) [29].

Data will be collected from students at the schools during school hours; this activity will take approximately 1 hour. Within 1 month, both parents and schoolteachers will complete a set of questionnaires on their children/pupils; each adolescent will be evaluated by a single schoolteacher. The parent questionnaire takes 25-30 minutes to complete, whereas the schoolteacher questionnaire takes 20-25 minutes.

**Table 1:** Measurement tools.

Categories and measures		Source
**Exposure variables**		
	Gambling Behavior Scale for Adolescents	Student
	Real-Money Games	Student
	Video-Gaming Scale for Adolescents	Student
	External perception of the adolescent’s gambling behavior and money availability	Parent/teacher
**Psychodiagnostic assessment**		
	Child Behavior Checklist	Parent
	Strength and Difficulties Questionnaire	Student/parent/teacher
	Teacher’s Report Form	Teacher
	Youth Self-Report	Student
**Social factors**		
	Loneliness Scale	Student
	Social Network	Student
**Personal factors**		
	Demographic, social, and environmental information	Student/parent
	Economic resources	Student

### Measurement

#### Demographic, Social, and Environmental Information

We will obtain information on the student’s sex, age, place of residence and its characteristics, family composition, parental education level, and parental job from the student assessment. We will obtain information on the student’s infancy from both student and parent assessments.

#### Strength and Difficulties Questionnaire

The Strength and Difficulties Questionnaire (SDQ) [30] is a 25-item questionnaire developed with reference to the main nosological categories recognized by the DSM-IV (also valid for the DSM-5) and describing positive and negative attributes of adolescents. Each item is scored on a 3-point scale: “Not true,” “Somewhat true,” or “Certainly true.” The items are divided into 5 subscales each with 5 items: emotional symptoms, behavioral problems, hyperactivity inattention, peer relationship problems, and prosocial behavior. It is also possible to assess chronicity, distress, burden to others, and social impairment. The SDQ provides scores for 3 dimensions of impact: perceived difficulties, impact score, and a burden rating. There are 3 versions of the SDQ: self-report, parent report, and teacher report version. The Italian version of the SDQ has good psychometric properties, with Cronbach α ranging from .73 to .89 [31].

#### Achenbach System of Empirically Based Assessment School-Age Forms

The Achenbach System of Empirically Based Assessment (ASEBA) [32,33] is an integrated system of multi-informant assessment of behavioral, emotional, and social problems and adaptive functioning in youth aged 11-18. Adolescents complete the *Youth Self-Report* (YSR; 119 items), parents complete the *Child Behavior Checklist* (CBCL; 120 items), and schoolteachers complete the *Teacher’s Report Form* (TRF; 120 items). Each questionnaire consists of a problem section with items rated as “Not True” (0), “Somewhat or Sometimes True” (1), or “Very or Often True” (2).

Each of these 3 questionnaires provides 8 syndrome scales: anxious/depressed, withdrawn/depressed, and somatic complaints (together: internalizing or emotional problems); rule-breaking behavior and aggressive behavior (together: externalizing or behavioral problems); and social problems, thought problems, and attention problems. Internalizing score, externalizing score, and total problems score can be calculated by summing up the problem items. Additionally, items from the YSR, CBCL, and TRF can be grouped into the following DSM-IV–oriented scales (still valid for the DSM-5): affective problems, anxiety problems, somatic problems, attention deficit/hyperactivity problems, oppositional defiant problems, and conduct problems [34]. The YSR shows good internal validity (Cronbach α=.71-.95) [35]. Cronbach α was high for the Broadband and the Total Problem scales, ranging from .83 to .91 for the CBCL [33]. The Internalizing scale can be derived from the first 3 syndrome scales (Cronbach α=.86), and the Externalizing scale from the last 2 (Cronbach α=.85). The Total Problems scale was determined by adding up the individual item scores (Cronbach α=.84) [36]. The TRF ranged from 0.86 to 0.94 [33].

#### Loneliness Scale

The Loneliness Scale (LS) [37] consists of 6 statements (rated as “yes,” “more or less,” or “no”) designed to measure emotional, social, and overall loneliness. Cronbach α was .92 [38].

#### Economic Resources

Three self-report questions about how often the adolescent receives money, how much, and from whom.

#### Social Network

Three self-report questions on the adolescent’s subjective perception of the reasons to use social networks.

#### Video-Gaming Scale for Adolescents

The Video-Gaming Scale for Adolescents (VGS-A) [39] is a self-report questionnaire that evaluates adolescents’ video game habits and pathological use of video gaming. The ﬁrst section consists of 3 unscored items investigating video game habits: the frequency with which they have played each of the various video game genres during the last year, the devices used to game and the time spent on each of them, and preferences about the use of online or offline gaming. The second section is composed of 9 items rated on a 3-point scale: “never” (0), “sometimes” (1), or “often” (2), which were developed to relieve the 9 DSM-5 diagnostic criteria of pathological gaming among adolescents. Pathological gaming is measured by taking into account the severity of each symptom described by the 9 items. Cronbach α for VGS-A was .71 [40].

#### Gambling Behavior Scale for Adolescents

The Gambling Behavior Scale for Adolescents (GBS-A) [41] is a self-report questionnaire that assesses gambling behavior among adolescents. The ﬁrst section consists of 4 unscored items investigating the frequency of participation in each of the different gambling activities (eg, card games, bets on sports games, lotteries) during the last year, the age at which gambling started, the existence of any gambling partners and the relative frequency of gambling with each of them, and the amount of money spent on each of the various gambling activities. The second section is composed of 9 items rated on a 3-point scale: “never” (0), “sometimes” (1), or “often” (2), which were developed to relieve the 9 DSM-5 diagnostic criteria of gambling disorder among adolescents. The GBS-A classifies the respondents as nonproblem gamblers, at-risk gamblers, or disordered gamblers. Cronbach α for GBS-A was .77 [42].

#### Real Money Games

A total of 16 self-report questions were developed to assess the frequency of and time spent in real money games (both online and offline) during the last year. These provide information on where money games are played, which devices are used to play money games, and the amount of money played.

#### Adults’ Perception of the Adolescent’s Gambling Behavior and Money Availability

Adults’ (both parents and schoolteachers) perceptions of their adolescent child/pupil’s involvement or noninvolvement in gambling behaviors and his/her money availability will be evaluated.

### Sample Size Estimation

The primary endpoint of the study is the prevalence of pathological gambling, pathological video game use, and emotional/behavioral problems in a sample of adolescent students attending upper-secondary schools. A sample size of 750 students produces a 2-sided 95% CI of 1.5%, 2%, and 2.5% when the prevalence is equal to 5%, 10%, and 15%, respectively.

Little is known from previous literature about risk factors for pathological gambling, pathological video game use, and emotional/behavioral problems in adolescent students attending upper-secondary schools. Assuming a prevalence of 15% [43] and considering a power (1-β) of 0.80 and a type I error (α) of .05, a sample of 750 students would allow the detection of an odds ratio of 2.2 for a binary risk factor present in 30% of the students. An adjustment was made because a multiple regression of the independent variable of interest on the other independent variables in the logistic regression obtained an R^2^=0.5. According to the “one in ten” rule of thumb, the proposed sample size ensures to fit a multivariate logistic regression model with up to 12 independent variables.

### Statistical Analysis Plan

The main characteristics of the sample will be summarized using absolute and relative frequencies for categorical variables and through mean or median and range, interquartile range, and standard deviation for continuous variables.

The prevalence of pathological gambling, pathological video game use, and emotional/behavioral problems will be quantified in terms of proportion (relative frequency) and its 95% CI.

We will use the chi-square test for categorical variables, or the Fisher exact test when expected frequencies are lower than 5, to assess differences among categorical variables. The normality of continuous variables will be assessed graphically using the quantile–quantile plot and through a nonparametric test (eg, the Shapiro–Wilk test). We will compare continuous variables among groups defined by a categorical variable using the *t* test when the normality assumption is met, or otherwise using the nonparametric rank-based Wilcoxon–Mann–Whitney *U* test. The strength of the correlation among continuous variables obtained from different informants (ie, students, parents, and teachers) will be evaluated using the Pearson linear correlation coefficient or the Spearman rank correlation coefficient. The Cohen kappa statistic and the Bland–Altman plot will be used to test concordance among categorical and continuous variables, respectively.

Multivariate clustering techniques (principal component analysis, multiple correspondence analysis, hierarchical clustering) will be used to identify distinct gambler and pathological video gamer profiles according to their gambling/gaming behaviors based on data collected through the questionnaires. We will use these advanced multivariate statistical techniques to reduce the size of the data by identifying a small number of underlying main components without losing too much information.

A multivariate logistic regression model combined with variable selection techniques will be used to identify risk factors associated with pathological gambling and pathological video gaming. The results will be presented in terms of odds ratios with their 95% CI.

We will consider a *P* value less than .05 as statistically significant.

### Patient and Public Involvement

Adolescent students, parents, or the teachers were not involved in the design, or conduct, or reporting, or dissemination plans of this research.

### Ethics Approval

Our protocol was reviewed and approved by the Institutional Ethics Board of the Spedali Civili of Brescia, Italy (resolution n. NP3662 f 28-01-2020). All procedures performed in this study are in accordance with the ethical standards of the Institutional Ethics Board of the Spedali Civili of Brescia and with the 1964 Declaration of Helsinki and its later amendments. We will obtain written consent from the parents, on their own behalf and on behalf of their children under 18, the schoolteachers, and from the adolescents of legal age. Students under 18 will be also asked for their consent.

### Availability of Data and Materials

Data sharing does not apply to this article as data sets will not be generated or analyzed in this article.

### Dissemination

We will publish all results in peer-reviewed international journals indexed in Web of Science or Scopus databases and present them at national and international conferences.

## Results

According to our sample size calculation, we expect that at least 793 adolescent students from 10 schools (5 middle schools and 5 high schools) located in Brescia, northern Italy, will participate in the study.

## Discussion

This study will quantify the prevalence of pathological gambling, pathological video game use, and emotional/behavioral problems in a sample of adolescent students attending upper-secondary schools in Brescia and its province, the second most populous province in Lombardy (northern Italy). This study will contribute to the implementation of a system for identifying pathological gambling and pathological video game use among adolescents, allowing for interventions aimed at improving adolescents’ financial, emotional, and social well-being. Furthermore, findings from this study will allow us to identify distinct profiles of gamblers and pathological video gamers that will contribute to setting up effective targeted prevention measures. Understanding the causes and impact of gambling and pathological video gaming on adolescents is a public health issue. We will present a report with the study results to the Osservatorio Provinciale del contrasto alle ludopatie e al gioco d’azzardo di Brescia [Provincial Observatory for the Prevention of Compulsive Gambling Disorders and Betting of the Lombardy region] and the Ufficio scolastico regionale per la Lombardia Ufficio IV Ambito Territoriale di Brescia [Regional School Office for Lombardy, IV District of Brescia], and the teaching staff of the schools and educational institutions involved. Additionally, a public conference will be organized as the project’s final step to inform students, parents, and the general population.

## Abbreviations

ASEBA: Achenbach System of Empirically Based Assessment
CBCL: Child Behavior Checklist
DSM: Diagnostic and Statistical Manual of Mental Disorders
GBS-A: Gambling Behavior Scale for Adolescents
SDQ: Strength and Difficulties Questionnaire
TRF: Teacher’s Report Form
VGS-A: Video-Gaming Scale for Adolescents
YSR: Youth Self-Report
